# Adipose and serum zinc alpha-2-glycoprotein (ZAG) expressions predict longitudinal change of adiposity, wasting and predict survival in dialysis patients

**DOI:** 10.1038/s41598-022-13149-6

**Published:** 2022-05-31

**Authors:** Gordon Chun-Kau Chan, Win Hlaing Than, Bonnie Ching-Ha Kwan, Ka-Bik Lai, Ronald Cheong-Kin Chan, Jeremy Yuen-Chun Teoh, Jack Kit-Chung Ng, Kai-Ming Chow, Winston Wing-Shing Fung, Phyllis Mei-Shan Cheng, Man-Ching Law, Chi-Bon Leung, Philip Kam-Tao Li, Cheuk-Chun Szeto

**Affiliations:** 1 Department of Medicine and Therapeutics, Prince of Wales Hospital, Carol & Richard Yu Peritoneal Dialysis Research Centre, The Chinese University of Hong Kong, Shatin, NT Hong Kong, China; 2 Li Ka Shing Institute of Health Sciences (LiHS), Faculty of Medicine, The Chinese University of Hong Kong, Shatin, Hong Kong, China; 3 Department of Anatomical and Cellular Pathology, The Chinese University of Hong Kong, Shatin, Hong Kong, China; 4 Department of Surgery, S.H. Ho Urology Centre, The Chinese University of Hong Kong, Shatin, Hong Kong, China

**Keywords:** Obesity, Nutrition, Nephrology, Renal replacement therapy, Peritoneal dialysis

## Abstract

There were limited data on adipose and serum zinc alpha-2-glycoprotein (ZAG) expression and its association with body composition in patients with advanced chronic kidney disease (CKD). This study aimed to quantify adipose and serum ZAG expression and evaluate their association with body composition and its longitudinal change, together with mortality in incident dialysis patients. We performed a single-center prospective cohort study. Patients who were planned for peritoneal dialysis were recruited. ZAG levels were measured from serum sample, subcutaneous and pre-peritoneal fat tissue obtained during peritoneal dialysis catheter insertion. Body composition and functional state were evaluated by bioimpedance spectroscopy and Clinical Frailty Scale respectively at baseline and were repeated 1 year later. Primary outcome was 2-year survival. Secondary outcomes were longitudinal changes of body composition. At baseline, the average adipose and serum ZAG expression was 13.4 ± 130.0-fold and 74.7 ± 20.9 µg/ml respectively. Both adipose and serum ZAG expressions independently predicted adipose tissue mass (ATM) (p = 0.001, p = 0.008, respectively). At 1 year, ATM increased by 3.3 ± 7.4 kg (p < 0.001) while lean tissue mass (LTM) remained similar (p = 0.5). Adipose but not serum ZAG level predicted change in ATM (p = 0.007) and LTM (p = 0.01). Serum ZAG level predicted overall survival (p = 0.005) and risk of infection-related death (p = 0.045) after adjusting for confounders. In conclusion, adipose and serum ZAG levels negatively correlated with adiposity and predicted its longitudinal change of fat and lean tissue mass, whilst serum ZAG predicted survival independent of body mass in advanced CKD patient.

## Introduction

Dialysis is one of the promising treatments that can effectively remove toxin, maintain homeostasis, and prolong life expectancy in patients with advanced chronic kidney disease (CKD). However, dialysis therapy induces protein loss to the dialysate, excessive calories uptake, inflammation, and catabolism. All these culminate obesity and muscle wasting, which are most apparent at the first year of dialysis initiation^1–4^. These longitudinal changes in body composition not only cause metabolic problems, but also affect survival^5–8^.

Adipose tissue is one of the largest organs in the body. It functions as the major body energy storage, and secretes a wide range of pro- and anti-inflammatory mediators, also known as adipokines^9^, which modulate multiple inflammatory pathways and maintain body homeostasis. Zinc alpha-2-glycoprotein (ZAG) is one of the identified adipokines that is highly expressed in blood and adipose tissues from patients with CKD^10–12^. It acts as a lipid mobilizing factor which promotes lipolysis, inhibits lipogenesis, and regulates secretion of other adipokines^13,14^. Therefore, it is often regarded as one of the key body composition regulators, mediating obesity and wasting. Conflicting results have been reported regarding the linkage between serum ZAG expression and body composition in patients with CKD^15–17^, which supports its intrinsic paracrine and autocrine action that cannot be fully reflected by its circulating level^14,18^. However, there are by far only very limited data that reported adipose ZAG expression and its clinical relevance in patients with CKD.

The objectives of our study are to quantify the ZAG expression at adipose and serum levels in advanced CKD patients, to explore their association with cross-sectional and longitudinal change of body composition, and to identify its prognostic value.

## Materials and methods

### Study design

This is a single center prospective cohort study. The study was approved by the Joint Chinese University of Hong Kong—New Territories East Cluster Clinical Research Ethics Committee (Reference Number CREC-2008.554). All study procedures were in compliant with the Declaration of Helsinki. Consecutive incident adult dialysis patients were recruited from 1 January 2011 to 31 December 2013 prior to peritoneal dialysis catheter insertion at Prince of Wales Hospital. Patients who have active malignancy, on anabolic steroids or corticosteroid therapy, and those who were previously on another modality of renal replacement therapy (e.g., hemodialysis, renal transplant) for more than 3 months were excluded.

Written informed consent was obtained before study enrollment. After enrollment, clinical and laboratory data were obtained by chart review. Comorbidity load was evaluated by the Charlson Comorbidity Index (CCI) and the nutritional state was measured by the comprehensive Malnutrition-Inflammation Score (MIS). All subjects received standard dietary counselling by experienced dietitians at the time of dialysis training. In general, they were advised to have energy intake 25 to 35 kcal/kg/day (depending on body mass index (BMI)), 25–30% of which is fat, and protein 1.0–1.2 g/kg/day.

### Body composition and functional state assessment

Basic anthropometric parameters including body weight, BMI (calculated as body weight divided by square of height), waist circumference (measured at mid-point between lowest lateral border of ribs and uppermost border of iliac crest), hip circumference (measured as widest portion of the buttocks), midarm circumference and triceps skinfold thickness were measured at baseline. Overweight, general, and central obesity were defined according to the WHO criteria on BMI and gender-specific median of waist circumference respectively.

Multi-frequency bioimpedance spectroscopy device (Body Composition Monitor [BCM], Fresenius Medical Care, Germany), was used to measure the volume of different body compartments^19^. Electrodes were attached to one hand and one foot with the patient in a supine position after drainage of dialysate. Extracellular water (ECW), intracellular water (ICW), lean tissue mass (LTM), and adipose tissue mass (ATM) were computed.

Frailty assessment was evaluated by retrospective review on clinical assessment forms and notes, as described previously^20^. Patients were classified into class 1 to 9 according to the Clinical Frailty Scale (CFS)^21^, and they were diagnosed as frail if they belonged to class 4 to 9.

Anthropometry, body composition and function state assessments were repeated 1 year later.

### Specimen collection

1–2 g of subcutaneous and pre-peritoneal adipose tissue samples were obtained during the insertion of peritoneal dialysis (PD) catheter (also known as Tenckhoff catheter) by mini laparotomy. The adipose tissue was then processed immediately and stored at − 80 °C overnight).

Serum samples were collected during the standard peritoneal equilibrium test (PET) session, which were performed around 4 weeks after dialysis catheter insertion, and when patient was stable, without concurrent peritonitis and in euvolemic state. The samples were sent to laboratory for processing immediately or stored in 4 °C overnight after collection.

### Detection of ZAG

The methods of RNA extraction have been described previously^20^. ZAG mRNA expression in the adipose tissue was measured by real-time quantitative polymerase chain reaction (RT-QPCR), using the Applied Biosystems Step One Plus system (Foster City, CA). Commercially available Taqman primers and probes, including 2 unlabeled PCR primers and 1 FAM™ dye labeled TaqMan® MGB probe, were used (all from Applied Biosystems). We used the phosphoglycerate kinase-1 (Applied Biosystems) as the housekeeping gene. Results were analyzed with Sequence Detection Software v2.0 (Applied Biosystems) and the relative quantification method by ∆∆Ct was applied for expression of targets in fold compared to the expression detected in samples from healthy subjects.

Serum ZAG level was measured by the commercially available ELISA kit (Alpha-2 Glycoprotein/Glycoprotein/ZAG/A2GP1 Human ELISA kit, BMS 2201, Invitrogen™, Carlsbad, CA) following the instructions from manufacturer. All assays were performed in duplicate. The detection limit of ZAG is 0.174 ng/ml.

### Outcome measures

All subjects were followed for a total of 3 years. The overall clinical management was decided by the attending clinician and was not affected by the study. The primary outcome measure was 2-year all-cause mortality following body composition re-assessment. The secondary outcomes were longitudinal changes of body composition.

### Statistical analysis

Statistical analysis was performed by SPSS for Windows software version 24 (SPSS Inc., Chicago). Descriptive data were presented as mean ± SD. Baseline clinical parameters were compared by Student’s t-test, chi-square test, and one-way analysis of variance (ANOVA), while the correlation was analyzed by Pearson and Spearman’s rank correlation as appropriate. The baseline and longitudinal change in body composition were compared among ZAG expression divided into tertiles. Multivariate linear regression models were then constructed to identify significant predictor of baseline and longitudinal change in body composition parameters. Kaplan Meier plots on ZAG expression in tertiles (low expression: 37.8–62.6 µg/ml; medium expression: 63.0–82.8 µg/ml; high expression: 83.0–149.1 µg/ml) were constructed and compared by log-rank test. Multivariate Cox proportional hazards models were constructed to further identify independent predictors of survival after adjustment of known potential confounders. The final p < 0.05 was considered as statistically significant. All probabilities were two-tailed.

## Results

### Patient characteristics

**Figure 1:**
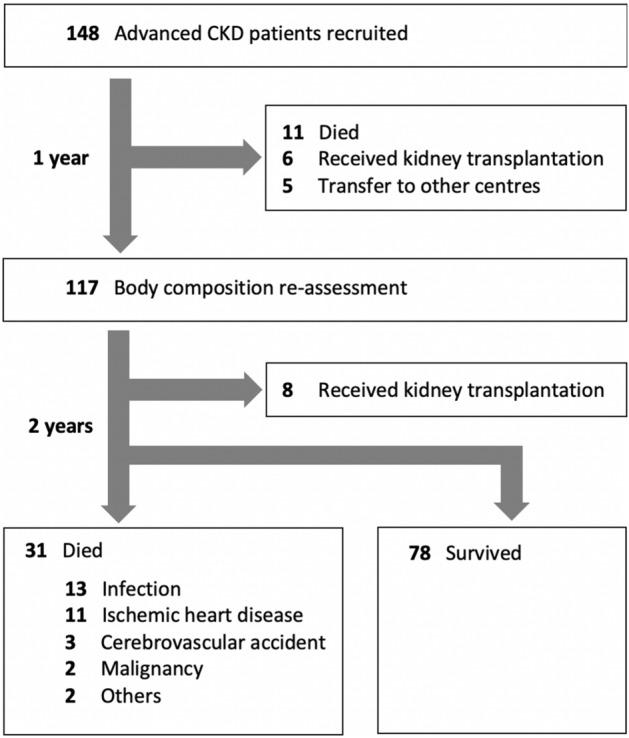
Flow chart.

148 incident dialysis patients were enrolled (Fig. 1). Their baseline clinical characteristics, and ZAG expression are summarized in Table 1. The mean age was 58.4 ± 11.3 years, and 111 (75%) were male. Serum ZAG level correlated with age (r = − 0.231, p = 0.006) and serum albumin (r = 0.27, p = 0.001), while adipose ZAG correlated with serum urea (r = 0.178, p = 0.03), residual renal function (r = − 0.233, p = 0.008) and serum lipids profile including total cholesterol (r = 0.175, p = 0.038), triglycerides (r = − 0.326, p < 0.001), high-density lipoprotein (r = 0.393, p < 0.001) and low-density lipoprotein (r = 0.177, p = 0.035). Their subsequent dialysis therapy including modality, dialysis adequacy and dialysate dextrose load were comparable between high and low adipose ZAG expression (Table 2).

**Table 1:** Clinical and biochemical characteristics.

Age	58.4 ± 11.3
Male, no. (%)	111 (75%)
**Primary renal disease, no. (%)**	
Diabetes mellitus	75 (50.7%)
Glomerulonephritis	33 (22.3%)
Hypertension	14 (9.5%)
Polycystic kidney disease	3 (2.0%)
Urological	6 (4.1%)
Others	2 (1.4%)
Unknown	15 (10.1%)
**Co-existing comorbidities, no. (%)**	
Ischemic heart disease	40 (27.2%)
Cerebrovascular accident	28 (19.0%)
Peripheral vascular disease	12 (8.2%)
Residual renal function (ml/min/1.73m^2^)	3.9 ± 2.7
Charlson comorbidity Index	6.1 ± 2.5
Malnutrition-inflammation Score	6.8 ± 3.7
Clinical Frailty Scale (CFS)	4.0 ± 1.3
Frail by CFS, No. (%)	39 (26.4%)
**Laboratory parameters**	
Urea (mmol/L)	30.7 ± 7.6
Creatinine (umol/L)	851 ± 268
Albumin (g/L)	35.3 ± 4.4
High sensitive C-reactive protein (mg/L)	12.0 ± 28.6
Total cholesterol (mmol/L)	4.5 ± 1.2
Triglycerides (mmol/L)	1.5 ± 0.9
High-density lipoprotein (HDL) (mmol/L)	1.3 ± 0.4
Low-density lipoprotein (LDL) (mmol/L)	2.6 ± 1.0
**Zinc alpha-2-glycoprotein (ZAG) expression**	
Adipose tissue ZAG (fold)	13.4 ± 130.0
Serum ZAG (microgram/ml)	74.7 ± 20.9

Data are expressed as mean ± standard deviation.

**Table 2:** Peritoneal dialysis therapy.

	Low adipose ZAG (n = 73)	High adipose ZAG (n = 73)	P-value
**Peritoneal transporter characteristics**			
D/P4	0.7 ± 0.1	0.7 ± 0.1	p = 0.3^a^
MTAC	10.3 ± 5.0	11.5 ± 5.1	p = 0.2^a^
Dialysis adequacy (Total Kt/V)	2.1 ± 0.7	2.0 ± 0.6	p = 0.2^a^
**Peritoneal dialysis modality**			p = 0.5^b^
CAPD, no. (%)	57 (78.1%)	60 (82.2%)	
Machine-assisted PD, no. (%)	16 (21.9%)	13 (17.8%)	
Icodextrin use no. (%)	18 (24.7%)	20 (27.4%)	p = 0.7^b^
Dextrose exposure (g/day)	107 ± 34	104 ± 42	p = 0.6^a^
Residual renal function (ml/min/1.73m^2^)	4.4 ± 2.7	3.4 ± 2.6	p = 0.03^a^
NPNA (g/kg/day)	1.1 ± 0.3	1.1 ± 0.2	p = 0.8^a^

*ZAG* zinc alpha-2-glycoprotein, *D/P4* dialysate-to-plasma ratio of creatinine at 4th hour, *MTAC* mass transfer area coefficient of creatinine, *CAPD* continuous ambulatory peritoneal dialysis, *NIPD* nocturnal intermittent peritoneal dialysis, *CCPD* continuous cycler peritoneal dialysis, *NPNA* normalized protein nitrogen appearance.

Data are expressed as mean ± standard deviation and compared by ^a^Student’s t-test and ^b^chi-square test.

There was no internal correlation between serum and adipose ZAG expressions (p = 0.1). There was also absence of significant correlation between serum ZAG and the product of adipose ZAG and all adiposity parameters including BMI (p = 1.0), waist circumference (p = 0.9), and ATM (p = 0.5).

### Baseline body composition

**Figure 2:**
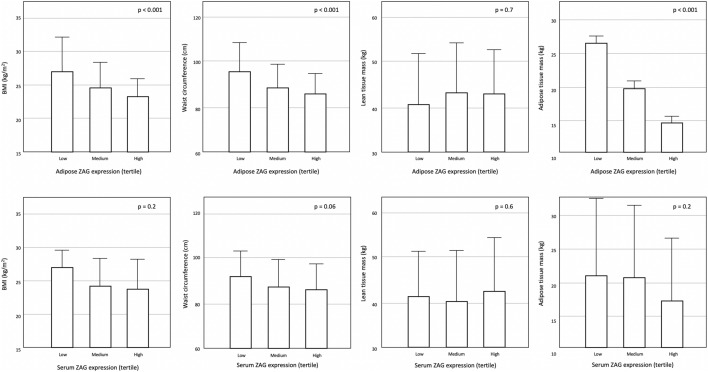
Baseline body composition parameters with adipose and serum ZAG expressions.

The baseline anthropometric and body composition parameters are summarized in Table 3. Adipose ZAG correlated with all body composition parameters, whereas serum ZAG correlated with waist, midarm circumference and ATM only (Supplementary Table 1). 13% and 24.5% were general and central obese respectively. BMI, waist circumference and ATM, but not lean tissue mass (LTM), significantly reduced in a stepwise manner along adipose and serum ZAG expressions (Fig. 2). In multivariate linear regression analysis, both adipose (unstandardized B: − 2.093, p = 0.001) and serum ZAG expressions (unstandardized B − 0.201, p = 0.008) predicted baseline ATM independently after adjustment for confounders including age, serum albumin, residual kidney function and MIS score.

**Table 3:** Anthropometry, body composition and functional state at baseline and at 1 year.

	Baseline	At 1 year	Change	P-value
**Anthropometry**				
Body weight (kg)	65.8 ± 14.5	67.7 ± 14.2	2.7 ± 5.6	p < 0.001
Body height (m)	1.6 ± 8.4	1.6 ± 8.3	− 0.1 ± 2.2	p = 0.643
Body mass index (kg/m^2^)	24.5 ± 4.3	25.2 ± 4.2	1.1 ± 2.2	p < 0.001
Waist circumference (cm)	88.7 ± 11.6	94.4 ± 17.2	4.0 ± 13.7	p = 0.022
Hip circumference (cm)	95.5 ± 9.1	95.7 ± 22.7	1.0 ± 25.3	p = 0.742
Midarm circumference (cm)	25.9 ± 3.0	/	/	/
Triceps skinfold thickness (mm)	9.8 ± 3.7	/	/	/
**Body composition**				
Lean tissue mass (kg)	41.5 ± 10.9	40.4 ± 11.4	− 0.5 ± 7.5	p = 0.505
Lean tissue index (kg/m^2^)	15.4 ± 3.3	15.0 ± 3.4	− 0.2 ± 2.8	p = 0.522
Adipose tissue mass (kg)	20.0 ± 11.1	23.0 ± 11.2	3.2 ± 7.3	p < 0.001
Fat tissue index (kg/m^2^)	7.4 ± 4.0	8.7 ± 4.2	1.2 ± 2.7	p < 0.001
Total body water (L)	38.4 ± 8.8	38.2 ± 8.5	− 0.2 ± 5.1	p = 0.682
Extracellular water (L)	19.3 ± 4.9	19.1 ± 4.5	− 0.2 ± 3.2	p = 0.468
Intracellular water (L)	19.1 ± 4.5	19.1 ± 4.6	0.02 ± 2.8	p = 0.943
**Functional state**				
Clinical frailty scale	4.0 ± 1.3	4.0 ± 1.3	0.05 ± 1.5	p = 0.686

Data are expressed as mean ± standard deviation and compared by Student’s t-test.

### Longitudinal change in anthropometry

With time, 11 (7.4%) patients died, 6 (4.1%) patients underwent renal transplantation, 9 (6.1%) patients were switched to hemodialysis, and 5 (3.4%) patients were transferred to other centers (Fig. 1). 117 patients underwent follow-up body composition assessment. Their BMI and waist circumference were increased by 2.01 ± 6.09 (p < 0.001) and 4.0 ± 13.7 cm (p = 0.02) respectively (Table 3). The longitudinal change in BMI significantly correlated with baseline serum albumin (r = 0.23, p = 0.01). In univariate model, serum albumin (unstandardized B 0.126, p = 0.014) predicted the longitudinal BMI change. However, the significance of serum albumin disappeared in multivariate model, in which age became the only independent predictor in this model (p = 0.03).

### Longitudinal change in body composition

**Figure 3:**
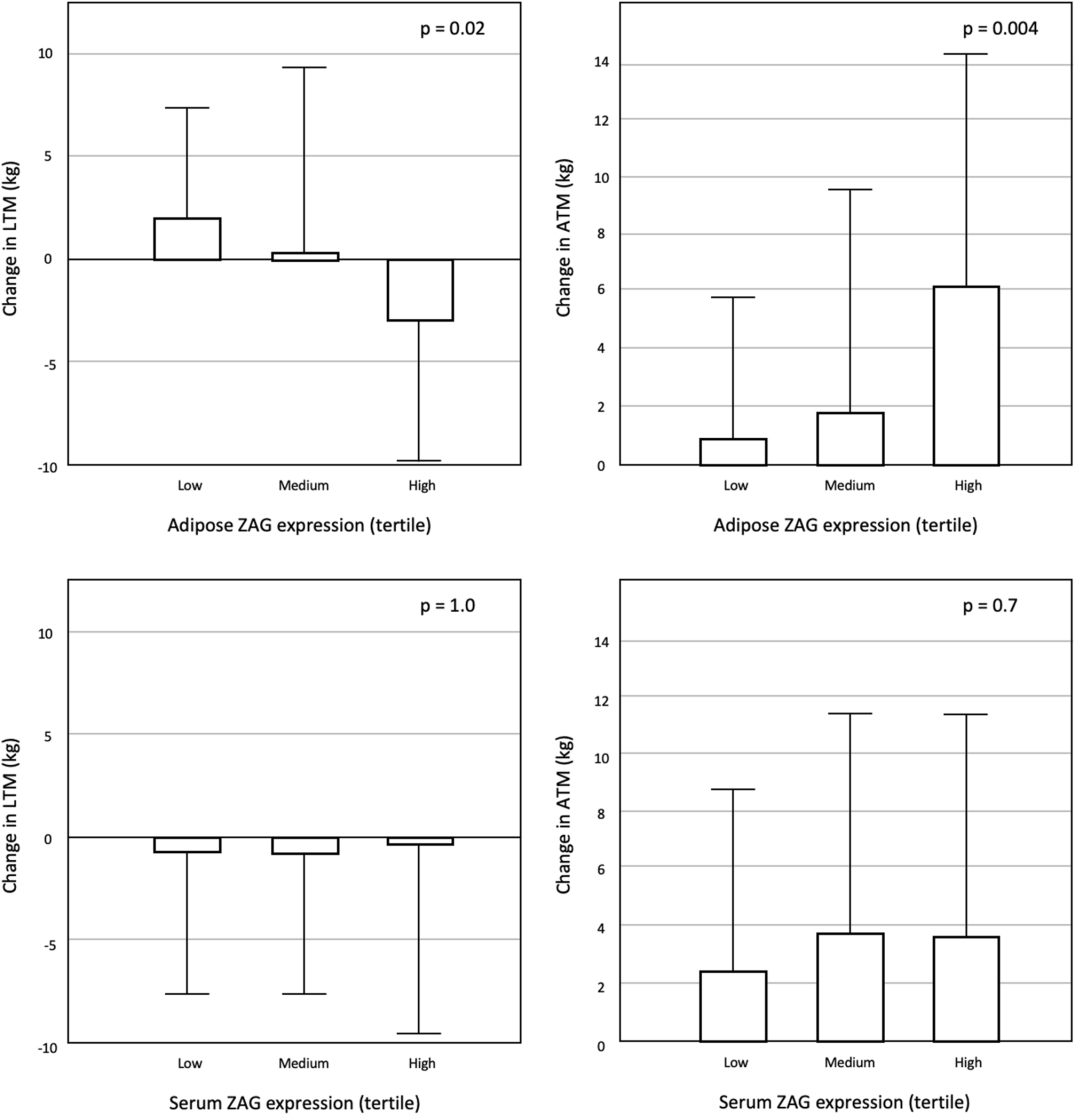
Change in lean and adipose tissue mass with adipose and serum ZAG expressions.

At 1 year, ATM increased by 3.3 ± 7.4 kg (p < 0.001), while their LTM only mildly reduced by 0.4 ± 7.6 kg (p = 0.5). Patients with higher adipose but not serum ZAG level tended to gain ATM and lose LTM with time (Fig. 3). Adipose ZAG level positively correlated with longitudinal change of ATM (r = 0.276, p = 0.004) and ICW (r = 0.286, p = 0.003), but inversely correlated with change of LTM (r = − 0.296, p = 0.002). In multivariate linear regression model, adipose ZAG expression remained as significant predictor of change in LTM, ATM and ICW (Table 4). In the same model, dialysis adequacy by total Kt/V also predicted ATM and ICW change. Serum albumin (unstandardized B 0.351, p = 0.040) predicted LTM change in univariate model, but its significance disappeared in multivariate model after adjustment for confounders. Dialysate dextrose load did not predict longitudinal change of ATM (p = 0.2) and LTM (p = 0.2).

**Table 4:** Multivariate linear regression analysis on change in body composition.

	LTM change		ATM change		ICW change	
	Unstandardized B (95% CI)	P-value	Unstandardized B (95% CI)	P-value	Unstandardized B (95% CI)	P-value
Adipose ZAG	− 1.30 (− 2.30–0.29)	p = 0.01	1.24 (0.36–2.12)	p = 0.007	− 0.34 (− 0.67–− 0.01)	p = 0.05
Total Kt/V	3.64 (0.33–6.96)	p = 0.03			1.37 (0.13–2.60)	p = 0.03

*BMI* body mass index, *LTM* lean tissue mass, *ATM* adipose tissue mass, *ICW* intracellular water, *ZAG* zinc alpha-2-glycoprotein, *CI* confidence interval.

Co-variates included in the models: age, serum albumin, total Kt/V, malnutrition-inflammation score (MIS).

### Survival

**Figure 4:**
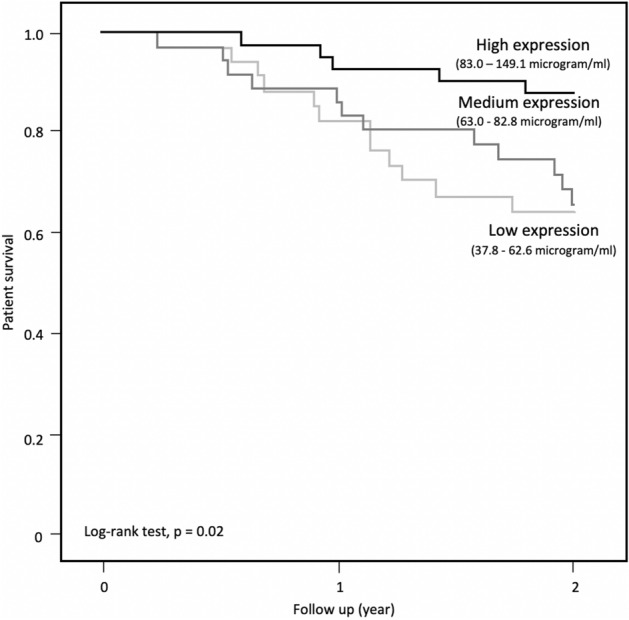
Kaplan Meier curve of survival.

Our patients were further followed for a total of 314.7 patient-years after body composition reassessments. During the period, 8 (6.8%) patients underwent kidney transplantation, and 31 (26.5%) patients died. The causes of death were infection (n = 13), ischemic heart disease (n = 11), cerebrovascular accident (n = 3), malignancy (n = 2) and others (n = 2). The average survival was 1.7 ± 0.6 years. Patients who died were older (p = 0.007) with a lower serum albumin level (p = 0.02). They also had a higher comorbidity load (p = 0.003) and were frailer (p < 0.001). Patients with lower serum ZAG expression experienced a stepwise significantly worse survival (log-rank test, p = 0.02) (Fig. 4). In multivariate model, serum ZAG level (p = 0.003), CFS (p < 0.001), baseline BMI (p = 0.04), LTM (p = 0.03) and ATM (p = 0.04) predicted all-cause survival (Table 5, Supplementary Table 2). Neither did longitudinal change of LTM and ATM predict survival in this model. Using the same group of covariates, serum ZAG (adjusted hazard ratio (AHR) 0.95, p = 0.045), CFS (AHR 3.40, p = 0.002), serum albumin (AHR 0.82, p = 0.04) and presence of ischemic heart disease (AHR 0.09, p = 0.02) independently predicted infection-related death, while presence of ischemic heart disease (AHR 3.65, p = 0.045) remained as the only predictor of cardiovascular-related death.

**Table 5:** Cox regression on survival.

	Univariate analysis		Multivariate analysis	
	Hazard ratio (95% CI)	P-value	Adjusted Hazard ratio (95% CI)	P-value
Serum ZAG	0.98 (0.96–1.00)	p = 0.015	0.97 (0.95–0.99)	p = 0.005
CFS	1.65 (1.29–2.11)	p < 0.001	1.73 (1.28–2.35)	p < 0.001
Baseline BMI	1.00 (0.92–1.08)	p = 0.9	1.29 (1.01–1.64)	p = 0.04
Baseline LTM	0.98 (0.94–1.01)	p = 0.17	0.93 (0.87–0.99)	p = 0.03
Baseline ATM	1.00 (0.97–1.04)	P = 0.8	0.91 (0.83–0.99)	p = 0.04
Age	1.05 (1.01–1.09)	p = 0.009		
Albumin	0.92 (0.86–0.99)	p = 0.03		
CCI	1.23 (1.06–1.41)	p = 0.005		

*CI* confidence interval, *ZAG* zinc alpha-2-glycoprotein, *CFS* Clinical Frailty Scale, *BMI* body mass index, *LTM* lean tissue mass, *ATM* adipose tissue mass, *CCI* Charlson Comorbidity Index.

Co-variates included in model: age, gender, residual renal function, albumin, high sensitive C-reactive protein, low density lipoprotein, CCI, presence of ischemic heart disease, CFS, baseline body mass index, adipose tissue mass (ATM) and LTM, absolute change of ATM and LTM, serum ZAG.

## Discussion

This study provides novel evidence of adipose and serum ZAG expression and its relationship with cross-sectional and longitudinal change in body composition and functional state in incident dialysis patients. In essence, adipose and serum ZAG level inversely correlated with adiposity, whilst patients with high adipose ZAG expression tended to gain fat and lose lean tissue with time on dialysis. Moreover, our result characterized the significance of serum ZAG level in survival prediction, which is independent from traditional risk factors like baseline and serial change in body composition. Here we provide evidence to the potential use of adipose and serum ZAG quantification from adipose and serum samples as biomarkers of adiposity and longitudinal change of fat and lean tissue, as well as to predict mortality in incident dialysis patients.

To date, limited data exist on correlation between body composition and ZAG expression at adipose tissue from human patients with CKD. There was only one published study that quantified adipose ZAG expression in 7 stage 5 CKD patients^11^. Here we report an absence of correlation between adipose and serum ZAG expression, which was illustrated in the past^11,14^. Such finding reinforces a key message that measurement of circulating ZAG level, as most published studies did, could not be used to deduce its expression at adipose tissue level. This also suggests the primary function of ZAG as an autocrine or paracrine rather than endocrine polypeptide^14,18^. Although ZAG is highly produced in adipose tissue^11^ which is extensively perfused by an extensive network of blood vessels, only a few adipokines like adiponectin and leptin that can flow freely and readily into the systemic circulation^22^. Moreover, the mode and route of metabolism could also influence the circulating level. By far there is no dedicated study that explored the mechanism of ZAG metabolism and elimination, though observational studies suggested a potential renal excretion route as plasma ZAG level increases when kidney function declines^10–12^, and its level decreases after patients regain renal excretory function through kidney transplantation^23^. Dialysis therapy itself may influence the circulating ZAG level^11^. However, the contributory role of renal clearance is less prominent in patients with advanced CKD who have very low renal excretory function, as reflected by the absence of correlation between serum ZAG level and both uremia and residual renal function from our results. Furthermore, we did not identify any correlation between serum ZAG and the product of adipose ZAG concentration and all adiposity parameters. Such finding supports the notion that adipose tissue is only one of the circulating ZAG sources. As a matter of fact, ZAG is also produced in the epithelial cells of various organs e.g., mammary gland, prostate, sweat gland, etc.^24^, and these may also contribute to the circulating level.

Our result expands the current evidence on the inverse relationship between adiposity and circulating ZAG level. While Leal et al.^25^ and Hosseinzadeh-Attar et al.^15^ used skinfold thickness and BMI respectively as proxy of adiposity, we used bioimpedance method to characterize fat tissue volume. However, inaccurate measurement of fat mass has been reported by using skinfold thickness and BMI^26,27^. In contrast, bioimpedance-based assessment is a reliable and sensitive tool to determine volume of different body compartments. It gives information on lean tissue mass, adipose tissue mass and hydration volume. Bioimpedance-based method is a practical, inexpensive tool that can achieve the substantial agreement with dual-energy x-ray absorptiometry (DXA)^26^, and it is currently a suggested method to assess body composition by the KDOQI guideline^28^.

Indeed, our dialysis patients gained a sizable amount of weight after dialysis initiation, which is predominantly contributed by fat mass gain, rather than lean tissue mass gain or overhydration. Such finding is in concordant with previous studies^29–31^, and this problem tends to be more apparent at the start of dialysis^30^. While diabetes mellitus, peritoneal dialysate glucose absorption^1^ predicted fat gain in peritoneal dialysis patients, we extend the understanding by illustrating adipose ZAG accelerated the process of adipose mass acquisition. Further study in animal model showed that zinc administration could induce adipose tissue hypertrophy^32^ and affect secretion of other adipokines^18^. The difference in change of body built between high and low ZAG level groups could be explained by regression to the mean phenomenon^33^, but the chance of such is low given the change in both LTM and ATM were all in one direction with just a difference in magnitude. Further study is warranted to identify whether ZAG or zinc-based treatment could revert the process of fat accumulation. Nonetheless, our patients with high adipose ZAG expression also experienced a greater loss in lean tissue mass. It could be explained by the fatty infiltration at the intermuscular region secondary to fat accumulation. This causes decline in muscle mass, quality and strength, which could eventually lead to reduction in physical performance, mobility, increase in fall risk and sequential development of frailty^34^. Moreover, ZAG positively correlates with both adipose triglyceride lipase (ATGL) and hormone-sensitive lipase (HSL), which play critical roles in myocyte apoptosis and proteosomal muscle degradation^35^ and result in cachexia and lean tissue wasting.

Our result reinforced the observation that patients with high ZAG expression have a better survival. In this aspect, ZAG plays an important role in the anti-inflammation and anti-fibrotic process^18,36^, as it reduces oxidative stress through down regulation of various pro-atherogenic factors production e.g., tumor necrosis factor-alpha (TNF-alpha), vascular cell adhesion molecule-1 (VCAM-1)^37^, etc. Together with its lipolytic effect, which results in a better lipid and glucose metabolism^15^, patients with high ZAG expression are less likely to develop coronary artery disease^38^. Likewise, ZAG also upregulates the activity of beta-2 adrenergic receptor^39^, which results in reduction in cardiac expression of type I, IV collagen and fibronectin and subsequent myocardial fibrosis. The use of beta-2 adrenergic receptor agonist has been proven to confer protective effect against cardiovascular complications through attenuation of monocyte activation, inflammatory and fibrotic responses in the heart^40^. The association with survival persisted after adjustment with baseline and trajectory of body composition after dialysis initiation. As baseline and trajectory of body composition, especially obesity, were identified as key factors for survival in dialysis patients^41,42^, this suggests these factors are likely merely proxies of ZAG expression.

Our reported results on serum ZAG with survival is different from that reported by Bouchara et al.^16^. Such discrepancy could be explained by several reasons. Firstly, the nature of subjects in the two studies are different. While Bouchara A’s study recruited prevalent hemodialysis (HD) patients, we recruited patients newly started on PD therapy. As a matter of fact, HD and PD patients represent two distinct groups of individuals since they have a different circulating ZAG profile^43^. Secondly, the timing of obtaining blood specimen (i.e., a pre-, mid-, or post- dialysis sample) was not clearly specified in Bouchara A’s study. In fact, serum ZAG fluctuates during HD. It was reported that pre-dialysis ZAG level differs from post-dialysis level^11^, and therefore interpretation of serum ZAG at different time point may lead to a different conclusion. Compared to HD, PD is characterized by a longer dialysis time with a continuous clearance of solute and toxins. Therefore, serum ZAG expression in PD should be more stable during a day between different time point. In addition to the traditional factors like age, gender, and albumin, we constructed a more comprehensive analysis model by adjusting our results for comorbidity load, frailty^44^, as well as the baseline and dynamic change of bioimpedance-derived body composition parameters^45^ to the regression model. These factors may account for the difference in the outcomes that we reported. In short, our result provides grounds supporting the use of serum ZAG quantification in mortality risk stratification.

Our study carries several limitations. Firstly, the power of our study is restricted by nature of single center study with a limited sample size, and we were unable to establish the causal relationship with outcome based on the intrinsic limitation of a cohort study. Secondly, the adipose tissue sample obtained during the operation may contain other cell types that may affect the ZAG assay. Since we obtained the serum sample approximately four weeks after peritoneal dialysis catheter insertion, there was a chance that dialysis therapy may affect ZAG expression, though the effect is expected to be minimal given a short duration of dialysis. Thirdly, we measured ZAG expression once only at baseline, thus we are unable to provide information on any longitudinal change in ZAG expression. Moreover, ZAG expression could be affected transiently by dietary habits, which were unmeasured in the current study. However, all our subjects received standard counselling on the proper amount of energy, fat, and protein intake by experienced dietitians at the start of study. We also reduced the confounding effects of such by adding metabolic and nutritional parameters such as albumin, lipids profile and BMI into the statistical analysis model. Furthermore, ZAG levels are also altered in acute stress conditions like sepsis and critical illnesses^46–48^, though we attempted to reduce the bias through exclusion of patients who were critically ill and unfit for peritoneal dialysis catheter insertion. Lastly, we did not evaluate other associated up- and down-stream biomarkers, therefore we are unable to infer the overall pathogenic pathway and establish the pathophysiological mechanisms with the clinical outcomes we studied.

In conclusion, adipose and serum ZAG levels significantly and positively correlated with adiposity in advanced CKD patients. Our finding also showed adipose ZAG predicted longitudinal change in adiposity and lean tissue wasting, which suggests that ZAG plays an important role in modulation of body composition. From the current study, we provided sufficient evidence to support the role of ZAG in survival prediction. Further observational and interventional studies are warranted to explore and establish to causal relationship and potential benefits of ZAG-based therapy.

## Supplementary Information

Supplementary Information 1.

Supplementary Information 2.

## Data Availability

Data described in the manuscript will be made available upon request to the corresponding author.
