# Supplementary material for: Adipose and serum zinc alpha-2-glycoprotein (ZAG) expressions predict longitudinal change of adiposity, wasting and predict survival in dialysis patients
Source: Sci Rep. 2022 May 31;12:9087. doi: 10.1038/s41598-022-13149-6 (PMC9158927; doi:10.1038/s41598-022-13149-6)
Supplement: Supplementary file 1 — Supplementary Information 1. [file 41598_2022_13149_MOESM1_ESM.docx]

# SUPPLEMENTARY MATERIALS

## Supplementary Table 1. Correlations between serum and adipose ZAG expression with baseline body composition parameters.

|  | Adipose ZAG | Serum ZAG |
| --- | --- | --- |
| Body weight | r = 0.232  p = 0.006 | r = -0.177  p = 0.4 |
| Body mass index | r = 0.352  p < 0.001 | r = -0.169  p = 0.5 |
| Waist circumference | r = -0.293  p < 0.001 | r = 0.232  p = 0.006 |
| Midarm circumference | r = -0.288  p < 0.001 | r = -0.178  p = 0.04 |
| Triceps skinfold thickness | r = -0.419  p < 0.001 | r = -0.106  p = 0.2 |
| Adipose tissue mass | r = -0.416  p < 0.001 | r = -0.191  p = 0.03 |
| Lean tissue mass | r = 0.048  p = 0.6 | r = 0.062  p = 0.5 |

ZAG, zinc alpha-2-glycoprotein.

Data were compared by Spearman's rank correlation coefficient.

## Supplementary Table 2. Cox regression analysis on all-cause survival.

|  | Univariate analysis | | Multivariate analysis | |
| --- | --- | --- | --- | --- |
|  | Hazard ratio  (95% CI) | P-value | Adjusted Hazard ratio  (95% CI) | P-value |
| Serum ZAG | 0.98  (0.96 – 1.00) | p = 0.015 | 0.97  (0.95 – 0.99) | p = 0.005 |
| CFS | 1.65  (1.29 – 2.11) | p < 0.001 | 1.73  (1.28 – 2.35) | p < 0.001 |
| Baseline BMI | 1.00  (0.92 – 1.08) | p = 0.9 | 1.29  (1.01 – 1.64) | p = 0.04 |
| Baseline LTM | 0.98  (0.94 – 1.01) | p = 0.17 | 0.93  (0.87 – 0.99) | p = 0.03 |
| Baseline ATM | 1.00  (0.97 – 1.04) | p = 0.8 | 0.91  (0.83 – 0.99) | p = 0.04 |
| Albumin | 0.92  (0.86 – 0.99) | p = 0.03 | 1.27  (1.00-1.63) | p = 0.05 |
| Age | 1.05  (1.01 – 1.09) | p = 0.009 | 0.98  (0.92 – 1.04 | p = 0.5 |
| CCI | 1.23  (1.06 – 1.41) | p = 0.005 | 1.05  (0.88 – 1.27) | p = 0.6 |
| Male | 1.06  (0.47 – 2.37) | p = 0.9 | 1.17  (0.37 – 3.70) | p = 0.8 |
| Residual renal function | 0.98  (0.85 – 1.12) | p = 0.7 | 1.10  (0.91 – 1.32) | p = 0.3 |
| hsCRP | 1.00  (0.98 – 1.01) | p = 1.0 | 0.99  (0.97 – 1.01) | p = 0.4 |
| LDL | 0.83  (0.57 – 1.20) | p = 0.3 | 0.96  (0.58 – 1.58) | p = 0.9 |
| Presence of IHD | 1.21  (0.57 – 2.58) | p = 0.6 | 0.68  (0.23 – 2.04) | p = 0.5 |
| Absolute change in ATM | 0.98  (0.93 – 1.03) | p = 0.5 | 1.01  (0.94 – 1.09) | p = 0.7 |
| Absolute change in LTM | 0.99  (0.95 – 1.04) | p = 0.8 | 1.01  (0.93 – 1.10) | p = 0.8 |

CI, confidence interval; ZAG, zinc alpha-2-glycoprotein; CFS, Clinical Frailty Scale; BMI, body mass index; LTM, lean tissue mass; ATM, adipose tissue mass; CCI, Charlson Comorbidity Index; hsCRP, high sensitive C-reactive protein; LDL, low density lipoprotein; IHD, ischemic heart disease.
